# Immune-mediated neurological syndromes in SARS-CoV-2-infected patients

**DOI:** 10.1007/s00415-020-10108-x

**Published:** 2020-07-30

**Authors:** Antoine Guilmot, Sofia Maldonado Slootjes, Amina Sellimi, Maroussia Bronchain, Bernard Hanseeuw, Leila Belkhir, Jean Cyr Yombi, Julien De Greef, Lucie Pothen, Halil Yildiz, Thierry Duprez, Catherine Fillée, Ahalieyah Anantharajah, Antoine Capes, Philippe Hantson, Philippe Jacquerye, Jean-Marc Raymackers, Frederic London, Souraya El Sankari, Adrian Ivanoiu, Pietro Maggi, Vincent van Pesch

**Affiliations:** 1 Department of Neurology, Cliniques Universitaires Saint-Luc, Université Catholique de Louvain, Hippocrate 10, 1200 Brussels, Belgium; 2 Department of Internal Medicine, Cliniques Universitaires Saint-Luc, Université Catholique de Louvain, Brussels, Belgium; 3 Department of Radiology, Cliniques Universitaires Saint-Luc, Université Catholique de Louvain, Brussels, Belgium; 4 Department of Biology, Cliniques Universitaires Saint-Luc, Université Catholique de Louvain, Brussels, Belgium; 5 Department of Intensive Care, Cliniques Universitaires Saint-Luc, Université Catholique de Louvain, Brussels, Belgium; 6 Department of Neurology, Clinique Saint-Pierre Ottignies, Ottignies-Louvain-la-Neuve, Belgium; 7 Department of Neurology, CHU UCL Namur Site Godinne, Yvoir, Belgium

**Keywords:** SARS-CoV-2, Cerebrospinal fluid, Anti-GD1b, Encephalitis

## Abstract

**Background:**

Evidence of immune-mediated neurological syndromes associated with the severe acute respiratory syndrome coronavirus (SARS-CoV-2) infection is limited. We therefore investigated clinical, serological and CSF features of coronavirus disease 2019 (COVID-19) patients with neurological manifestations.

**Methods:**

Consecutive COVID-19 patients with neurological manifestations other than isolated anosmia and/or non-severe headache, and with no previous neurological or psychiatric disorders were prospectively included. Neurological examination was performed in all patients and lumbar puncture with CSF examination was performed when not contraindicated. Serum anti-gangliosides antibodies were tested when clinically indicated.

**Results:**

Of the 349 COVID-19 admitted to our center between March 23rd and April 24th 2020, 15 patients (4.3%) had neurological manifestations and fulfilled the study inclusion/exclusion criteria. CSF examination was available in 13 patients and showed lymphocytic pleocytosis in 2 patients: 1 with anti-contactin-associated protein 2 (anti-Caspr2) antibody encephalitis and 1 with meningo-polyradiculitis. Increased serum titer of anti-GD1b antibodies was found in three patients and was associated with variable clinical presentations, including cranial neuropathy with meningo-polyradiculitis, brainstem encephalitis and delirium. CSF PCR for SARS-CoV-2 was negative in all patients.

**Conclusions:**

In SARS-Cov-2 infected patients with neurological manifestations, CSF pleocytosis is associated with para- or post-infectious encephalitis and polyradiculitis. Anti-GD1b and anti-Caspr2 autoantibodies can be identified in certain cases, raising the question of SARS-CoV-2-induced secondary autoimmunity.

## Introduction

A broad spectrum of neurological symptoms occurring during coronavirus disease 2019 (COVID-19) infection, such as anosmia, headache, impaired consciousness, cerebrovascular disease and skeletal muscle injury, have been promptly reported [1]. Since then, two cases of possible encephalitis and para- or post-viral immune-mediated polyneuropathies, including one case associated with the presence of anti-GD1b IgG antibodies have been described in COVID-19 patients [2–5]. However, little is known about the immune-mediated neurological syndromes associated with severe acute respiratory syndrome coronavirus-2 (SARS-CoV-2) infection. Recent studies reported almost no evidence of CSF inflammation in severely affected COVID-19 patients [6, 7]. Only a few cases have been reported with positive SARS-CoV-2 PCR in CSF [8]. In this pilot study, we describe the neurological manifestations, serological and CSF findings of SARS-CoV-2-infected patients.

## Methods

From March 23rd through April 24th 2020, consecutive COVID-19 positive patients with neurological manifestations were prospectively enrolled at Cliniques universitaires Saint-Luc (Brussels, Belgium) and affiliated hospitals (CHU UCL Namur site Godinne and Clinique Saint-Pierre Ottignies). A positive diagnosis of COVID-19 infection was established either by SARS-CoV-2 PCR assay of nasopharyngeal swabs or a positive SARS-CoV-2 IgG serology.

Patients were not included if (1) neurological presentation was isolated anosmia and/or non-severe headache (as these symptoms were considered to be not sufficiently suggestive of CNS involvement), and (2) they had a history of previous neurological or psychiatric disorders (to avoid the confounding factor of a fortuitous exacerbation). All patients underwent neurological examination. Serum anti-gangliosides antibodies (anti-GM1, anti-GM2, anti-GD1a, anti-GD1b and anti-GQ1b IgG) were initially tested in patients with suspicion of peripheral nerve involvement, and then systematically in subsequent patients with ataxia or features suggestive of brainstem involvement; only high-titer IgG (> 1/100) are reported here. Onconeuronal antibodies (anti-Tr, anti-GAD65, anti-Zic4, anti-Titin, anti-SOX1, anti-recoverin, anti-Hu, anti-Yo, anti-Ri, anti-Ma2/Ta, anti-CV2/CRMP5, anti-amphiphysin) and anti-neuronal antibodies (anti-NMDAR, anti-LGI1, anti-CASPR2, anti-GABARB1, anti-DPPX, anti-AMPAR) were tested when encephalitis was suspected. Lumbar puncture (LP) with CSF study was performed in all patients, when not contraindicated. Brain imaging and serological workup were acquired according to the clinical care needs of patients. Patients were categorized as having severe or non-severe COVID-19 infection, as previously described [1]. All patients were treated with hydroxychloroquine for 6 days according to institutional guidelines. The study was approved by the local ethics committee of each medical center and informed consent was obtained from all subjects/next-of-kin.

## Results

### Study sample and demographics

During the inclusion period, a total of 349 COVID-19 patients were admitted. Among those, 15 patients (4.3%) had neurological manifestations and fulfilled the study inclusion/exclusion criteria. Demographics, comorbidities and neurological features for severe and non-severe COVID-19 patients are summarized in Table 1. The onset of neurological symptoms occurred before or without development of respiratory symptoms in three patients (20%). Nevertheless, two of them had digestive symptoms now also considered as common symptoms of the disease. The delay between initial COVID-19 symptoms and neurological presentation is specified for each patient in Table 2.

**Table 1:** Demographic and clinical characteristics of patients with neurological manifestations according to severity of COVID-19 infection

	All patients	Severe	Non-severe
Participants, No.	15	9	6
Women, No.	3	2	1
Age, median (range), years	62 (37–84)	62 (54–84)	62 (37–80)
Comorbidities, No.			
Hypertension	11	9	2
Diabetes	5	3	2
Cardiovascular disease	5	4	1
Malignancy	5	3	2
Chronic kidney disease	4	4	–
Neurological manifestations, No.			
Delirium	5	4	1
Neuropsychiatric	3	1	2
Coma	2	2	–
Acute cerebrovascular disease	3	2	1
Cranial neuropathy	2	–	2
Associated seizures	2	1	1
Associated anosmia	2	–	2
Disease onset			
Neurological disease as first or isolated presentation	3	1	2

Neuropsychiatric alludes to the following symptoms: paranoia, hallucination, irritability, anxiety; cranial neuropathy: polyneuritis cranialis, ophtalmoparesis; associated seizures: patients who presented seizures as additional neurological manifestation; associated anosmia: patients who presented anosmia as additional neurological manifestation

**Table 2:** Neurological presentations, serological and CSF findings in COVID-19 patients

Patient	Age	Severity	Neurological presentation	Neuro onset delay (days)	CSF > 5 WBCs/μL	CSF protein levels (mg/dl)	CSF/serum albumin	OCB	Serum and CSF onconeural and anti-neuronal antibodies	Serum anti-GD1b IgG titer
1	37	Non-severe	Cranial neuropathy*	10	+	51	7.3^†^	Mirror	NA	> 1/100
2	40	Non-severe	Cranial neuropathy	5	−	36	NA	−	NA	NA
3	62	Severe	Coma	21	−	32	7.4^†^	Mirror	−	> 1/100
4	66	Severe	Coma	21	−	45	8.7^†^	−	−	−
5	80	Non-severe	Neuropsychiatric**	NA	+	46	6.1	CSF-specific^††^	Anti-Caspr2	NA
6	62	Severe	Neuropsychiatric**	16	−	51	12.4^†^	Mirror	−	NA
7	54	Non-severe	Neuropsychiatric	5	−	18	2.4	Mirror	−	−
8	71	Non-severe	Delirium***	5	−	32	6.1	−	−	> 1/100
9	60	Severe	Delirium	7	−	35	9.1^†^	−	NA	NA
10	66	Severe	Delirium	5	−	18	3.4	Mirror	NA	NA
11	81	Severe	Delirium	4	NA	NA	NA	NA	NA	NA
12	84	Severe	Delirium	2	NA	NA	NA	NA	NA	NA
13	58	Severe	Acute cerebrovascular disease	21	−	52	NA	NA	NA	NA
14	74	Non-severe	Acute cerebrovascular disease	NA	−	51	9.9^†^	Mirror	NA	NA
15	54	Severe	Acute cerebrovascular disease	NA	−	11	NA	−	NA	NA

Cranial neuropathy: polyneuritis cranialis, isolated ophtalmoparesis; neuropsychiatric: paranoia, hallucinations, irritability, anxiety; severity: as described in Metlay JP. et al., Am J Respir Crit Care Med., 2019; neuro onset delay: delay between initial COVID-19 symptoms (cough, fever, myalgia, dyspnea, digestive symptoms) and neurological presentation; WBCs: CSF white blood cells; OCB: oligoclonal bands; mirror: IgG oligoclonal bands with the same pattern in CSF as in serum; CSF-specific: CSF-specific IgG oligoclonal bands

+ : present; − : absent; NA: not available

*With cauda equina radiculitis

**With associated seizures

***With associated involuntary movements and ataxia

^†^CSF/serum albumin values above the median level for age, according to Hegen H. et al., Clin Chem Lab Med, Feb 2016; 54(2):285–92

^††^Presence of anti-Caspr2 antibodies in serum and in CSF

Serum anti-gangliosides antibodies were tested in 5 patients, serum onconeuronal and anti-neuronal antibodies were tested in 6 patients, while CSF samples were obtained in 13 patients (LP was contraindicated in 2 patients because of anticoagulation). Median delay between the onset of neurological symptoms and the LP was 3 days (range 1–32).

### Clinical, serological and CSF findings

The patients’ neurological presentations with their respective serological and CSF correlates are summarized in Table 2.

**Fig. 1:**
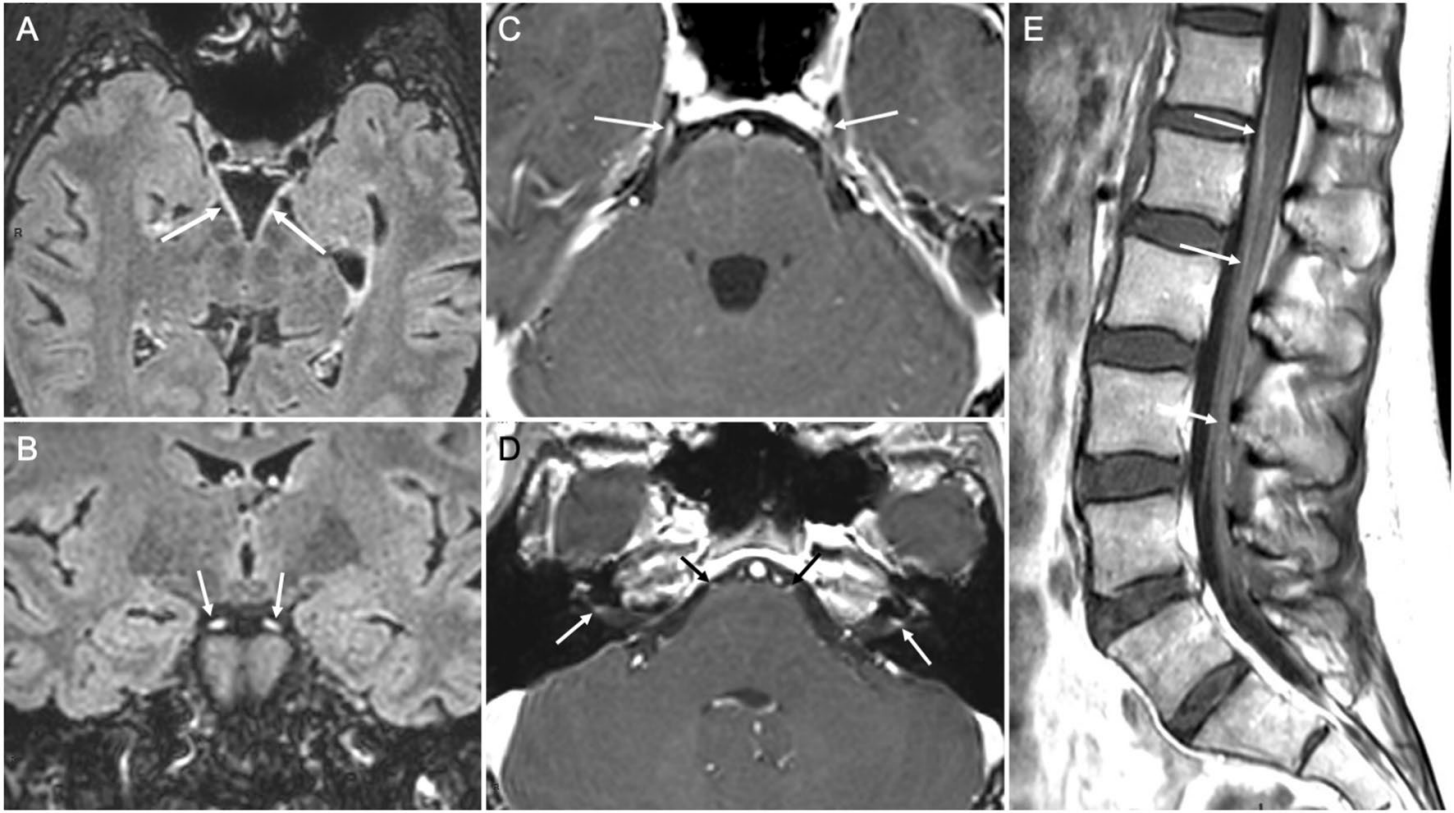
Brain and spinal cord MRI of a COVID-19 patient with meningo-polyneuritis. Thirty-seven-year-old woman who presented with cauda equina syndrome and multiple cranial neuropathies, 10 days after the onset of a non-severe SARS-CoV-2 infection (cough, pyrexia, myalgia, headache and vomiting but without dyspnea). Upon admission, she had no respiratory symptoms. Axial (**a**) and coronal (**b**) post-contrast T2 Fluid-attenuated inversion recovery (FLAIR) MRI demonstrated thickened and abnormally hyperintense III cranial nerves (arrows). Axial post-contrast T1-weighted images showed **c** abnormal bilateral enhancement of the cisternal segments of cranial nerve V (mainly of the Gasser’s ganglions; arrows), and **d** abnormal bilateral enhancement of the initial segment of nerve VI (black arrows) and of the meatal segment of nerve VII (white arrows). Post-contrast sagittal T1-weighted images of the lumbar spinal cord **e** showed abnormal periconal enhancement of the pia-mater (upper arrow) together with clumping and enhancement of the roots of the horse tail (lower arrows)

Two patients presented cranial nerve involvement. The first one (Patient 1) presented unilateral facial palsy, hemifacial paresthesia, bilateral hearing loss and paresthesia in lower limbs*,* with a history of cough, pyrexia, myalgia, headache and vomiting 10 days before. MRI showed multiple cranial nerve involvement and cauda equina enhancement (Fig. 1). Initial CSF examination showed 101 cells/μL (95% lymphocytes) without other abnormality; CSF studies from a second LP 12 days later showed 28 cells/μL (90% lymphocytes) and an elevated albumin quotient (Qalb). Infectious workup (including hemoculture, urinalysis with bacterial culture, Streptococcus pneumoniae antigen and Legionella pneumophila antigen; nasal swab for influenza A and B; serologies for EBV, CMV, HIV, Chikungunia, Dengue, Zika, syphilis, Borrelia; CSF bacterial culture and CSF multiplex PCR for enterovirus, HSV1 and 2, VZV, CMV, HHV6, human parechovirus, Escherichia coli, Haemophilus influenzae, Listeria monocytogenes, Neisseria meningitides, Streptococcus agalactiae, Streptococcus pneumoniae, Cryptococcus neoformans) was negative. Serum anti-gangliosides antibodies testing showed high-titer anti-GD1b IgG. After completion of the diagnostic workup, the patient was treated with 64 mg methylprednisolone for 7 days and improved gradually.

The other patient (Patient 2) presented with partial left oculomotor nerve III palsy 5 days after a febrile episode, without any respiratory symptoms. Brain MRI and CSF examination were normal. Antiganglioside antibody screening was not performed. The patient spontaneously improved.

Two patients (Patients 3 and 4) developed a comatose state. Patient 3 presented ophthalmoplegia, palatal myoclonus, neck stiffness and areflexic flaccid tetraplegia upon withdrawal of a 3-week-long sedation in the Intensive Care Unit (ICU). He had been previously admitted to the hospital with fever, cough, delirium and orthostatic hypotension, with a brutal worsening of his respiratory symptoms a few days later. Patient 4 presented to the hospital with delirium, reversal of circadian rhythm and digestive symptoms (nausea, vomiting, anorexia, constipation). In the next days, she developed agitation and hallucinations, and 3 weeks later neck stiffness, diffuse myoclonus, bilateral ophthalmoplegia, palatal tremor, apnea and coma. The patient was admitted to the ICU for a 1-week neurological surveillance. Brain MRI were unremarkable in both patients. Both LPs showed an elevated Qalb without pleocytosis. Serum anti-gangliosides antibodies testing showed high-titer anti-GD1b IgG in Patient 3 but not in Patient 4. Both patients were treated with intravenous immunoglobulin therapy and have been improving gradually at the time of writing.

Patient 5 presented a 3-week history of asthenia and weight loss, followed by several episodes of paroxysmal dysarthria, one tonic–clonic seizure followed by persistent neuropsychiatric symptoms (visual hallucinations, short-term memory disturbance and anxiety). No respiratory symptoms occurred. Two CSF examinations were performed 1-week apart and both showed 9 cells/µL (100% lymphocytes), CSF-specific IgG oligoclonal bands (OCB) on the second LP, sterile culture and negative PCR for infectious agents including Herpes Simplex and SARS-CoV-2. Continuous EEG-monitoring showed generalized slowing. Brain MRI was normal. Anti-contactin-associated protein 2 (Caspr2) IgG antibodies were positive both in the serum and CSF. Intravenous methylprednisolone therapy followed by plasmapheresis was initiated at the time of writing and epilepsy has not recurred under levetiracetam therapy.

Two other patients had neuropsychiatric symptoms (Patients 6 and 7). Patient 6 presented aggressiveness and paranoia followed by temporal status epilepticus 15 days after the onset of respiratory symptoms (cough, dyspnea and fever). Brain CT showed no acute lesion/process. CSF analysis showed increased Qalb. He was admitted to the ICU and required sedation for a month. At the time of writing, he was admitted to the neurological rehabilitation unit. The other patient presented behavioral changes, irritability and paranoia, within days of a dry cough, myalgia and headache. No fever was recorded. EEG showed generalized slowing. Brain CT and CSF were normal. His neuropsychiatric symptoms were still present at discharge after resolution of the respiratory illness. The patient declined any neurological follow-up. Metabolic tests, onconeuronal and anti-neuronal antibodies panels were negative in both patients.

Five patients (Patients 8, 9, 10, 11 and 12) had early-onset delirium without evident cause (i.e., no fever, metabolic disorder, hypoxemia). Patient 8 also presented akathisia, choreiform involuntary movements of the upper limbs and gait ataxia. In this patient, serum anti-gangliosides antibodies testing showed high-titer anti-GD1b IgG. CSF analysis was available in three patients and showed elevated Qalb in one (Patient 9). EEGs showed generalized slowing and brain CT was normal in all cases. Two patients were later admitted to the ICU in relation to their respiratory symptoms: Patient 10 stayed 1 week without the need of mechanical ventilation, while Patient 11 died shortly after ICU admission.

Three patients had acute cerebrovascular disease during the course of their respiratory illness. Two had ischemic strokes (Patients 13 and 14): one with an intracardiac thrombus and the other with patent foramen ovale and concomitant deep venous thrombosis. On CSF examination, one had an elevated Qalb. The third (Patient 15) had pulmonary embolism treated with low molecular weight heparin fractions with close therapeutic monitoring and presented multiple bilateral hematomas and subarachnoid hemorrhage.

Finally, PCR for SARS-CoV-2 on the CSF was negative in all patients.

## Discussion

In agreement with the available literature, our study confirms that viral genome is frequently undetectable by PCR in the CSF of SARS-CoV-2-infected patients [1]. However, we found CSF patterns consistent with blood-CSF barrier dysfunction and meningeal inflammation, such as elevated Qalb and/or lymphocytic pleocytosis.

We observed CSF lymphocytic pleocytosis in two cases: one with anti-Caspr2-associated limbic encephalitis and the other with para-infectious polyradiculitis. Autoimmune encephalitis (such as anti-NMDAR encephalitis) can occur following herpes simplex virus infection [9]. However, the pathophysiological role of viral infections in limbic encephalitis remains controversial. Noteworthily, in our SARS-CoV-2-positive patient with anti-Caspr2-associated encephalitis, no evidence of direct CNS infection nor underlying neoplasm was present. Autoimmune encephalitis was suspected in two other patients with neuropsychiatric symptoms and/or seizure but could not be proven. Para- or post-infectious polyradiculitis have been described in COVID-19 patients, but never associated with significantly increased CSF WBCs [2, 3]. In this context, our patient’s CSF pleocytosis, surprisingly associated with the presence of anti-GD1b IgG antibodies, clinical-imaging evidence of multiple cranial nerves and cauda equina inflammation suggest an immune reaction triggered by SARS-CoV-2.

Two COVID-19 patients with unexplained coma had clinical features highly suggestive of brainstem involvement associated with flaccid tetraplegia, suggesting potential concomitant peripheral nerve involvement. In both, LP demonstrated mild blood-CSF barrier dysfunction without pleocytosis. Serum anti-GD1b IgG were detected in one patient, a finding described in patients with Bickerstaff brainstem encephalitis and COVID-19-associated Miller Fisher syndrome [3, 10].

Interestingly, three patients in our study had high-titer serum IgG antibodies targeting the GD1b ganglioside. However, clinical presentations were highly variable, including cranial neuropathy with meningo-polyradiculitis, brainstem encephalitis and delirium with akathisia, choreiform involuntary movements, raising the question of whether these are truly pathogenic in all cases. GD1b is one of the most represented gangliosides in nerve tissues, but has been less studied than its GQ1b and GM1 counterparts [10]. Anti-GD1b antibodies have been described in the clinical spectrum of Guillain–Barré syndrome, Miller Fisher syndrome and Bickerstaff brainstem encephalitis, and are associated with more severe disease and slower recovery [11, 12]. More recently, anti-GD1b IgG antibodies were reported in a COVID-19 patient with Miller Fisher syndrome [3]. How anti-ganglioside autoimmunity arises in SARS-CoV-2 infection remains unclear. Structural and molecular modelling studies suggest that SARS-CoV-2 binds to respiratory tract gangliosides through its spike protein [13]. This association between COVID-19 and anti-GD1b-mediated autoimmunity has potential therapeutic implications (i.e., corticotherapy, intravenous immunoglobulins, plasmapheresis).

Three patients in our study had acute cerebrovascular disease associated with normal CSF findings or only mild blood-CSF barrier dysfunction. Whether stroke in COVID-19 patients may be partially immune-mediated is debatable. A recent case report described the presence of antiphospholipid antibodies in a SARS-CoV-2 infected patient with multifocal ischaemic stroke, even though a possible causative role remains uncertain [14].

This study has some limitations. First, our cohort size is rather small, therefore precluding a robust evaluation of the incidence of each presented neurological syndrome and CSF findings. Furthermore, the etiopathological role of SARS-CoV-2 in the observed neurological manifestations and laboratory abnormalities cannot be assessed. It cannot be excluded that some of the observed neurological syndromes, such as encephalopathy, may have resulted at least in part from our patients’ comorbidities. Such a confounding causality could only be demonstrated by pathological analysis.

In conclusion, our data suggest that CSF lymphocytic pleocytosis and/or blood-CSF barrier dysfunction is associated with para-infectious encephalitis and polyradiculitis in SARS-CoV-2 infected patients. Anti-GD1b and anti-Caspr2 autoantibodies can be identified in certain cases, raising the question of SARS-CoV-2-induced secondary autoimmunity [15]. In clinically relevant presentations, a thorough screening for autoantibodies should be initiated, as this may lead to therapeutic implications in some patients.

## Data Availability

PM and VvP had full access to all the data in the study and take responsibility for the integrity of the data and the accuracy of the data analysis.
